# FOXM1 and NFκB Form a Positive Feedback Loop to Promote Cell Growth and Drug Resistance in Mantle Cell Lymphoma

**DOI:** 10.3390/cells15090776

**Published:** 2026-04-25

**Authors:** Yujie Zhang, Yuqi Song, Meaad Almowaled, Chuquan Shang, Leizhao Hua, Irwindeep Sandhu, Anthea Peters, Michael P. Chu, Peng Wang, Raymond Lai

**Affiliations:** 1 Department of Physiology, School of Basic Medical Science, Nanjing Medical University, Nanjing 211166, China; zhangyujie@njmu.edu.cn; 2 Department of Laboratory Medicine and Pathology, University of Alberta, Edmonton, AB T6G 2R3, Canada; song23@ualberta.ca (Y.S.); chuquan@ualberta.ca (C.S.); leizhao@ualberta.ca (L.H.); 3 College of Applied Medical Sciences, King Saud bin Abdulaziz University for Health Sciences, Jeddah 22384, Saudi Arabia; mowaledm@ksau-hs.edu.sa; 4 Division of Hematology, Department of Medicine, University of Alberta, Edmonton, AB T6G 2R3, Canada; irwindee@ualberta.ca (I.S.); anthea.peters@albertahealthservices.ca (A.P.); mpchu@ualberta.ca (M.P.C.); 5 Department of Oncology, Cross Cancer Institute, Edmonton, AB T6G 1Z2, Canada

**Keywords:** mantle cell lymphoma, FOXM1, NFκB, positive feedback loop

## Abstract

**Highlights:**

**What are the main findings?**

- FOXM1 is upregulated in MCL and it correlates with a worse clinical outcome.
- FOXM1 and NFκB form a positive feedback loop.

**What are the implications of the main findings?**

- Inhibiting FOXM1 decreases the growth and drug resistance of MCL cells.
- FOXM1 represents a potential therapeutic target in MCL.

**Abstract:**

Mantle cell lymphoma (MCL) is an aggressive B-cell non-Hodgkin lymphoma characterized by the *t(11;14)(q13;q32)* cytogenetic abnormality and cyclin D1 overexpression. We have found evidence that Forkhead box M1 (FOXM1), a transcription factor with oncogenic potential, is important in the pathogenesis of MCL. Relatively high levels of FOXM1 proteins were detectable in all six MCL cell lines examined. By immunohistochemistry, we consistently found a subset of FOXM1-positive cells in MCL tumors. Analysis of two Gene Expression Omnibus (GEO) datasets from MCL patients showed that elevated *FOXM1* levels significantly correlate with a worse clinical outcome. In MCL cell lines, inhibition of FOXM1 using thiostrepton or shRNA effectively triggered apoptosis and significantly reduced cell growth. FOXM1 forms a positive feedback loop with NFκB in MCL cells. Specifically, inhibition of FOXM1 dramatically decreased the protein level/transcription activity of p65, while enforced FOXM1 expression upregulated p65 and downregulated IκBα, a key NFκB inhibitor. Conversely, curcumin-mediated NFκB inhibition decreased the protein level/DNA binding of FOXM1, while transduction of a constitutively active IKKα construct into MCL cells significantly dampened the inhibitory effects of thiostrepton. Confocal microscopy revealed that FOXM1 and p65 colocalize with each other. In conclusion, FOXM1 and NFκB work collaboratively in promoting the growth and drug resistance of MCL, and FOXM1 may be a potentially useful therapeutic target.

## 1. Introduction

Mantle cell lymphoma (MCL) is a type of aggressive B-cell non-Hodgkin lymphoma characterized by the *t(11;14)(q13;q32)* cytogenetic abnormality and cyclin D1 overexpression [1]. When treated with conventional combination chemotherapy, MCL patients had a median survival of approximately 3 years, although this clinical outlook has been significantly improved with the advent of novel therapeutics such as Bruton’s tyrosine kinase (BTK) inhibitors [2]. Nonetheless, disease relapse remains to be a significant clinical challenge. In recent years, there has been extensive research to identify the key pathogenetic factors in MCL. In this regard, deregulations of several cellular signaling pathways have been recognized to be important, as summarized in a recent review [3].

NFκB is one of the first signaling pathways that is implicated in the pathogenesis of MCL [4]. It has been reported that NFκB is constitutively active in MCL, and it promotes tumor growth by upregulating various anti-apoptotic genes and facilitators of cell-cycle progression [5,6]. More recent research has revealed that NFκB promotes oncogenesis and chemoresistance in MCL via its interactions with other signaling pathways [5,7]. For instance, NFκB promotes TLR4 signaling, which increases the growth of MCL cells and facilitates their evasion from immune surveillance [8]. In other studies, it was found that NFκB can promote the tumorigenesis of MCL by interacting synergistically with the PI3K/Akt signaling pathway [5,7,9,10]. NFκB has cross-talks with the Notch signaling pathway, and activation of this pathway (i.e., induced by mutations of the *Notch* gene) has been shown to contribute to the activation of B-cell receptor signaling and correlate with aggressive clinical behaviors [11]. Most recent research has highlighted the role of NFκB in conferring resistance to BTK inhibitors [5].

Forkhead box M1 (FOXM1) is a member of the Fox family of transcription factors which share the highly conserved winged helix DNA binding domain (also called the forkhead domain) [12]. It has been recognized as an oncoprotein that is highly expressed in many forms of cancer [12,13]. Functionally, FOXM1 plays a critical role in promoting cell cycle progression by transcriptionally regulating the expression of key cell cycle regulators such as cyclin B1 and CDC25B [14]. In addition, FOXM1 has been implicated in tumor invasion and metastasis by regulating the expression of various epithelial–mesenchymal transition-related genes and matrix metalloproteinase genes [15,16]. By regulating the expression of various genes involved in DNA repair and cell survival, FOXM1 contributes to other hallmarks of cancer including enhanced angiogenesis, resistance to DNA damage, and maintenance of stemness [13,17,18]. The biological significance of FOXM1 has been demonstrated in various solid cancer models, exemplified by the observation that inhibition of FOXM1 using pharmacologic agents such as thiostrepton can effectively suppress tumor growth [19]. In hematologic cancers, it has been shown that FOXM1 can exert oncogenic effects in acute lymphoblastic leukemia [20,21,22], myeloma [23], diffuse large B cell lymphoma [24], acute myeloid leukemia [25] and ALK-positive anaplastic large cell lymphoma [26]. Experimental data from studies of FOXM1 knockout mice is also in support of the notion that FOXM1 is a useful anti-cancer target. The biological and clinical significance of FOXM1 has not been extensively studied in MCL, although data suggesting that FOXM1 is oncogenic in MCL was included in a poster presentation [27].

In this study, we examined the clinical and biological significance of FOXM1 in MCL cell lines and tumors. Our findings showed that FOXM1 exerts potent oncogenic effects in MCL cells, and high levels of FOXM1 significantly correlate with a worse clinical outcome. Furthermore, our investigations have revealed a novel positive feedback loop formed by FOXM1 and NFκB in MCL.

## 2. Materials and Methods

### 2.1. Cell Culture

MCL cell lines (including MAVER-1, Z-138, Mino, JeKo-1, SP-53, and REC-1) and Lenti-X 293T cells (Dharmacon, Cambridge, UK) were cultured in RPMI-1640 or high-glucose DMEM (Gibco, Waltham, MA, USA), respectively, supplemented with 10% FBS and 1% penicillin-streptomycin (Gibco) at 37 °C with 5% CO_2_, as described previously [28].

### 2.2. Cell Viability Assay and Chemicals

Trypan blue stain (Gibco) and CCK8 (RayBiotech, Norcross, GA, USA) were used to quantify the viable cells. Inhibitory concentration 50% (IC50) was calculated using GraphPad Prism (version 10.1.0, GraphPad Software, San Diego, CA, USA). Curcumin, thiostrepton, and doxycycline were purchased from Cayman Chemical (Ann Arbor, MI, USA), Selleck Chemicals (Houston, TX, USA), and Thermo Fisher Scientific (Cleveland, OH, USA), respectively.

### 2.3. Western Blot Analysis and Co-Immunoprecipitation

Cell lysates were prepared using RIPA buffer or a Cell Lysis Reagent (Sigma-Aldrich, St. Louis, MO, USA). Equal amounts of protein were subjected to SDS-PAGE and immunoblotting. For co-immunoprecipitation (Co-IP), lysates were incubated with specific antibodies and protein A/G agarose beads (Santa Cruz Biotechnology, Dallas, TX, USA), followed by washing and Western blotting (Supplemental Materials).

### 2.4. Immunohistochemistry and Immunofluorescence

Immunohistochemical analysis was performed as previously described [29]. An anti-FOXM1 mouse antibody (Santa Cruz Biotechnology, 1:50) was used. Heat-induced epitope retrieval was performed by microwaving tissue sections in Tris-EDTA buffer (pH 9.0) for 15 min. Diaminobenzidine (Dako, Santa Clara, CA, USA) was used as chromogen. Immunofluorescence staining was conducted as previously described [30] and is detailed in Supplemental Materials.

### 2.5. Ethics Statement

The use of MCL patient samples was approved by the Institutional Research Ethics Committee of the University of Alberta (#Pro00062737).

### 2.6. Luciferase Assay

The empty vector luciferase reporter and FOXM1 luciferase reporter were constructed using the pGL4.10 backbone, kindly provided by Dr. Cater J Barger and Dr. Adam Karpf from the University of Nebraska Medical Center [31]. The pNiFty-Luc plasmid, harboring NFκB binding sites, was purchased from Invivogen (San Diego, CA, USA) (detailed in Supplemental Materials).

### 2.7. Nuclear/Cytoplasmic Fractionation and DNA Pull-Down

Subcellular fractionation was performed using an NE-PER Kit (Thermo Fisher Scientific). DNA pull-down assays were performed as previously described [26]. Briefly, 400 μg of protein was incubated with 4 μg biotinylated oligonucleotide probes containing the FOXM1 or p65 consensus binding sequence (Integrated DNA Technologies, Coralville, IA, USA) (detailed in Supplemental Materials).

### 2.8. Bioinformatic Analysis

The gene expression profiles (GSE10793 and GSE93291) and patient survival data were obtained from two separate Gene Expression Omnibus (GEO) datasets [32,33]. A total of 194 MCL samples were selected to analyze the expressions of FOXM1 and related genes. The NFκB pathway gene sets were derived from the KEGG database. Survival data were used to evaluate the prognostic value of FOXM1 and MKI67. The low- and high-gene-expression groups were defined using the median expression value (50%) as the cutoff (applied separately within each dataset). Overall survival (OS) was used as the clinical endpoint, and survival analyses were performed as described in Section 2.9. Multivariate models included FOXM1 and MKI67 as covariates. Proportional hazards assumptions were verified prior to analysis.

Single-cell RNA sequencing (scRNA-seq) datasets (GSE184031 and GSE303064) were analyzed using the Seurat R package (version 5.3.1, https://CRAN.R-project.org/package=Seurat (accessed on 22 April 2026)). After performing quality control, data normalization, and scaling, we performed principal component analysis (PCA) for dimensionality reduction. Unsupervised clustering was conducted using a graph-based clustering approach, and uniform manifold approximation and projection (UMAP) was used for visualization of cellular heterogeneity. Differential gene expression between groups (primary vs. relapse) was assessed using the Wilcoxon rank-sum test. *FOXM1* and *MKI67* expression levels were analyzed using feature plots and violin plots. High-expressing cells were defined as the top 20% of expressing cells based on expression quantiles, and co-expression patterns were used to identify double-high cell populations in each dataset. Cell-type annotation was performed based on canonical marker genes to identify major immune and stromal cell populations, including B cell-enriched clusters.

Data processing was performed using RStudio (version 2025.09.2+418, Posit Software, Boston, MA, USA) and GSEA (version 4.2.1, Broad Institute, Cambridge, MA, USA), with figures generated using GraphPad Prism and Adobe Illustrator 2020 (version 24.0, Adobe Inc., San Jose, CA, USA).

### 2.9. Statistical Analysis

Statistical analyses were performed using the SPSS software (Version 25.0, SPSS Inc., Chicago, IL, USA). Survival differences were assessed using Kaplan–Meier analysis with the log-rank test. Univariate and multivariate Cox proportional hazards regression models were used to calculate hazard ratios (HRs) and 95% confidence intervals (CIs). The proportional hazards assumption was tested prior to model fitting. For in vitro experiments, one-way or two-way analysis of variance (ANOVA) was used for multi-group comparisons, as appropriate, based on the experimental design. Student’s *t*-test was used for comparisons between two groups. Dose–response curves were analyzed using nonlinear regression to calculate half-maximal inhibitory concentrations (IC50). All tests were two-tailed, and *p* < 0.05 was considered statistically significant. Data are presented as mean ± standard deviation (SD). Figures were generated and assembled using GraphPad Prism and Adobe Illustrator software.

## 3. Results

### 3.1. FOXM1 Expression in MCL Cell Lines and Tumors

**Figure 1:**
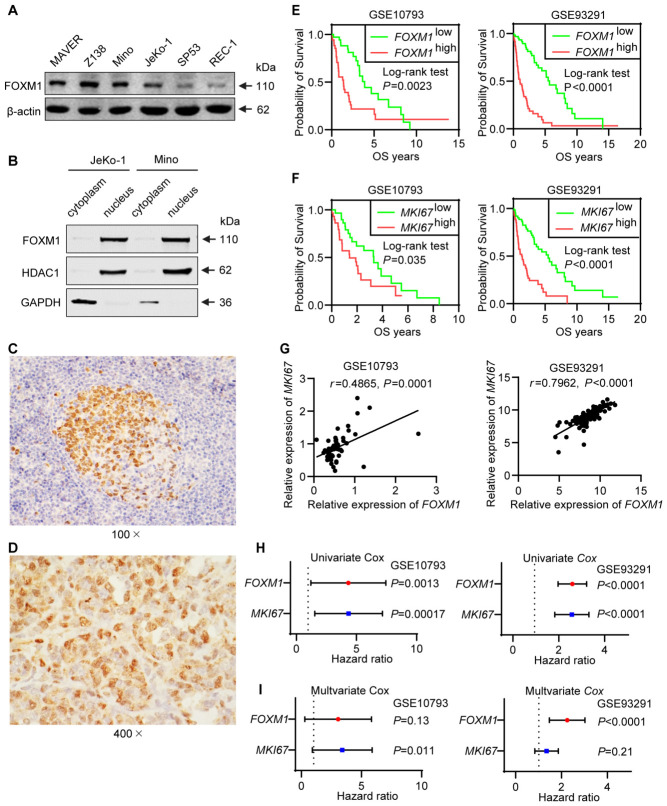
Forkhead box M1 (FOXM1) is highly expressed in the nuclei of MCL cells and has prognostic significance independent of MKI67. (**A**) Western blot analysis for FOXM1 expression in 6 MCL cell lines. β-actin was used as the loading control. (**B**) Cytoplasmic/nucleus fractionation was performed using JeKo-1 and Mino cells. HDAC1 (a nuclear marker) and GAPDH (a cytoplasmic marker) were used to assess the efficiency of the fractionation. (**C**,**D**) Expression of FOXM1 detectable by immunohistochemistry in a reactive tonsil (100×) and in a case of MCL (400×). In the reactive tonsil, FOXM1 expression is found largely in the dark zones of the germinal centers; mantle zones and interfollicular T-cells were largely negative. FOXM1 was strongly expressed in the nuclei of a subset of lymphoma cells. (**E**,**F**) Kaplan–Meier survival analysis of two independent Gene Expression Omnibus (GEO) cohorts (GSE10793 and GSE93291). Patients are dichotomized into high- and low-expressing groups using the median expression level of *FOXM1* (E) and *MKI67* (F) as the cut-off. Differences in the survival between the two groups are assessed using the log-rank test. (**G**) *FOXM1* expression correlates significantly with *MKI67* expression in both cohorts. Spearman’s correlation analysis revealed a significant association (GSE10793: *r* = 0.4865, *p* = 0.0001, *n* = 71; GSE93291: *r* = 0.7962, *p* < 0.0001, *n* = 123). (**H**,**I**) Univariate and multivariate Cox regression analysis is performed using the two patient cohorts (GSE10793, *n* = 71; GSE93291, *n* = 123). *FOXM1* and *MKI67* are evaluated for their association with the Overall survival (OS). In GSE10793, *FOXM1* has a hazard ratio (HR) of 3.57 (95% CI 1.64–7.75, *p* = 0.0013) in univariate analysis and a HR of 2.22 (95% confidence interval (CI) 0.80–6.16, *p* = 0.13) in multivariate analysis. *MKI67* has a HR of 3.73 (95% CI 1.88–7.42, *p* = 0.00017) and a HR of 2.78 (95% CI 1.26–6.13, *p* = 0.011) in univariate and multivariate analysis, respectively. In GSE93291, *FOXM1* has a HR of 2.54 (95% CI 1.99–3.24, *p* < 0.0001) in univariate analysis and a HR of 2.16 (95% CI 1.52–3.06, *p* < 0.0001) in multivariate analysis; *MKI67* has a HR of 2.49 (95% CI 1.86–3.34, *p* < 0.0001) and a HR of 1.28 (95% CI 0.87–1.89, *p* = 0.21) in univariate and multivariate analysis, respectively.

To survey FOXM1 protein expression in MCL, we performed Western blotting using a panel of six MCL cell lines, including MAVER-1, Z-138, Mino, JeKo-1, SP-53, and REC-1. As shown in Figure 1A, the FOXM1 protein band located at 110 kDa was readily detectable in all cell lines tested. Using nuclear/cytoplasmic fractionation, we found that FOXM1 is largely localized to the nuclei of MCL cells (Figure 1B). The HDAC1 and GAPDH bands were used to assess the efficiency of our nuclear/cytoplasmic fractionation. The expression of FOXM1 was then examined in 15 formalin-fixed, paraffin-embedded MCL tumors using immunohistochemistry; a reactive tonsil was used as the control group (Figure 1C,D). FOXM1 expression was consistently detectable in the nuclei of MCL cells, although the percentage of strongly positive cells varied from case to case.

### 3.2. FOXM1 Is a Prognostically Significant Marker for MCL Patients

To assess the clinical significance of FOXM1 in MCL patients, we correlated the gene expression level of *FOXM1* and the clinical outcome in two publicly available GEO datasets, GSE10793 and GSE93291, which were derived from two separate cohorts of 71 and 123 previously untreated MCL patients, respectively. As shown in Figure 1E, we found a highly significant inverse correlation between *FOXM1* and the OS in both cohorts (Log rank test, *p* = 0.0023 and <0.00010, respectively). As shown in Figure 1F, the gene expression level of *MKI67* also significantly correlates with a worse clinical outcome in these two cohorts (Log rank test, *p* = 0.035 and <0.00010, respectively), and these results regarding the prognostic significance of *MKI67* are consistent with those of a previously published report [34]. Since the gene expression level of *MKI67* significantly correlates with that of *FOXM1* (Figure 1G), we assessed if the prognostic value of *FOXM1* is independent of that of *MKI67*. As shown in Figure 1H,I, univariate and multivariate Cox regression analysis have revealed that the prognostic value of *FOXM1* is independent of that of *MKI67*.

**Figure 2:**
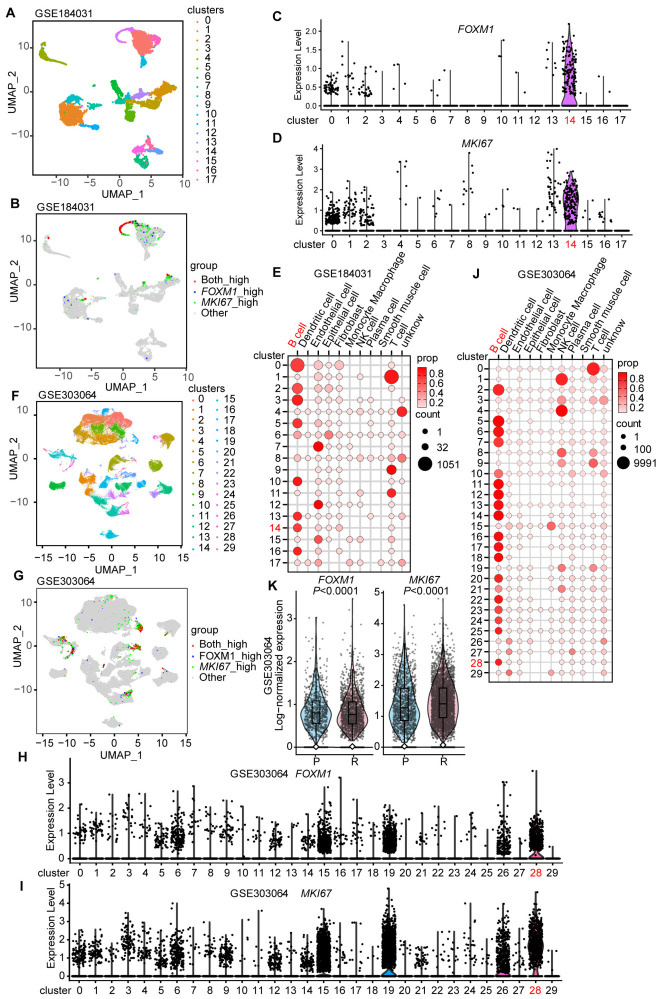
Single-cell transcriptomic analysis of *FOXM1* and *MKI67*. (**A**) uniform manifold approximation and projection (UMAP) of all cells from 4 MCL patients included in GSE184031 after unsupervised clustering shows intra-tumoral heterogeneity, with 18 clusters (0–17). (**B**) Feature plots of *FOXM1* and *MKI67* in GSE184031 highlights the co-expression of high level of *FOXM1* and *MKI67* in cluster 14. (**C**,**D**) Violin plots of *FOXM1* and *MKI67* across clusters in GSE184031; cluster 14 is the *FOXM1*/*MKI67* double high-expressing cluster. Clusters with significantly enriched *FOXM1* or *MKI67* expression were highlighted in color for visual emphasis. (**E**) Cell-type composition of clusters in GSE184031; cluster 14 is enriched for B cell-associated transcriptional signatures. (**F**) UMAP of all cells in GSE303064 after unsupervised clustering shows intra-tumoral heterogeneity, with 30 clusters (0–29). (**G**) Feature plots of *FOXM1* and *MKI67* in GSE303064 highlights the dual high expression in cluster 28. (**H**,**I**) Violin plots of *FOXM1* and *MKI67* across clusters in GSE303064; cluster 28 is the *FOXM1*/*MKI67* double high-expressing cluster. Clusters with significantly enriched *FOXM1* or *MKI67* expression were highlighted in color for visual emphasis. (**J**) Cell-type composition of clusters in GSE303064; cluster 28 is enriched for B cell-associated transcriptional signatures. (**K**) *FOXM1* and *MKI67* expression in primary (P) versus relapse (R) cells in GSE303064. Differences are analyzed using a two-tailed Wilcoxon rank-sum test, showing significant upregulation of both genes in relapse cells (*p* < 0.001).

We then performed single-cell transcriptomic analysis using two previously published MCL datasets (GSE184031 and GSE303064), which represent results from 4 MCL patients and 11 patients (some with paired initially diagnosed tumors and relapse tumors), respectively. As shown in Figure 2A, UMAP visualization of all cells in the GSE184031 dataset after unsupervised clustering highlights a high degree of intra-tumoral heterogeneity with respect to the expressions of *FOXM1* and *MIKI67*, and the feature plots reveal prominent co-enrichment of *FOXM1* high-expressing cells and *MKI67* high-expressing cells in cluster 14; this finding is further highlighted in the violin plots of *FOXM1* expression and *MKI67* expression across all clusters (Figure 2B–D). Cell-type composition analysis indicates that cluster 14 (highlighted in red) is enriched for B cell-associated transcriptional features (Figure 2E). Similar analyses using the GSE303064 dataset show a similar pattern, with cluster 28 identified as the *FOXM1*/*MKI67* double high-expressing cluster, which is also enriched for B cell-associated transcriptional features (Figure 2F–J). Furthermore, comparison of primary and relapse tumor samples in GSE303064 reveals that the expressions of *FOXM1* and *MKI67* are significantly higher in relapse cells (Wilcoxon rank-sum test, Figure 2K, *p* < 0.001), suggesting that the double *FOXM1*/*MKI67* high expressing cluster may play a key role in the disease relapse of MCL.

### 3.3. FOXM1 Promotes Cell Growth and Inhibits Apoptosis in MCL

**Figure 3:**
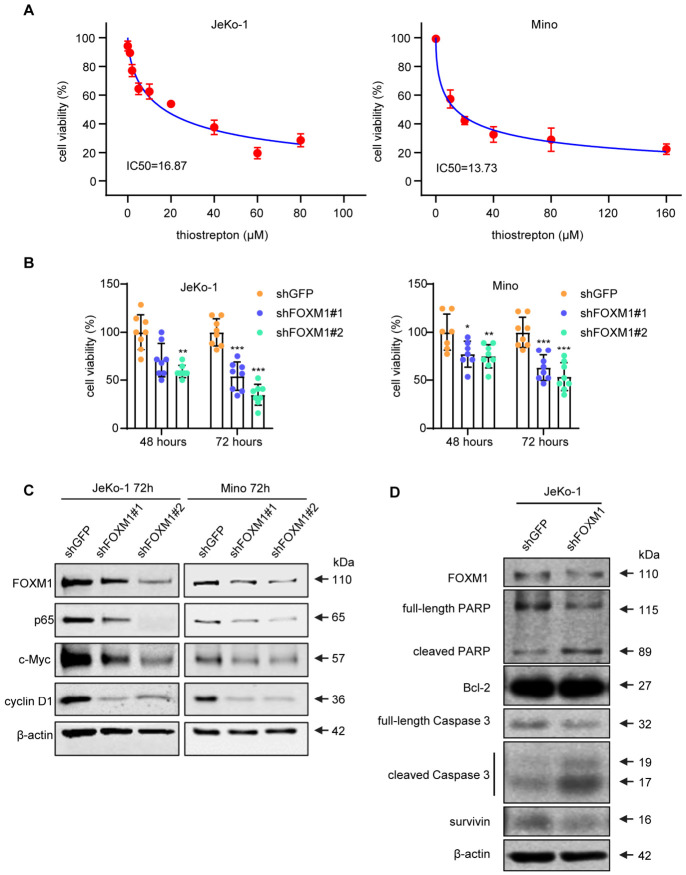
FOXM1 promotes cell growth and inhibits apoptosis in MCL. (**A**) Cell viability assays of JeKo-1 and Mino cells treated with thiostrepton for 24 h showed a dose-dependent inhibition of cell growth in both cell lines. Data are presented as mean ± SD (*n* = 4). Dose–response curves were analyzed using nonlinear regression to calculate the IC50 values (variable slope, four-parameter logistic model). (**B**) Cell viability assay of two MCL cell lines treated with *FOXM1* shRNA showed a significant reduction in cell growth (mean ± SD, *n* = 7, one-way analysis of variance (ANOVA) with Dunnett’s test vs. shGFP; * *p* < 0.05, ** *p* < 0.01, *** *p* < 0.001). (**C**) Western blot analysis using MCL cells treated with *FOXM1* shRNA showed substantial downregulations of p65, c-Myc and cyclin D1. (**D**) Western blot analysis using JeKo-1 cells treated with *FOXM1* shRNAs for 72 h showed dramatic reductions in Bcl-2 and survivin, and a marked increase in PARP/caspase 3 cleavage. β-actin was used as the loading control.

We then assessed the biological significance of FOXM1 in MCL by performing in vitro studies using two MCL cell lines, JeKo-1 and Mino. As illustrated in Figure 3A, the addition of thiostrepton, a widely studied pharmacologic inhibitor for FOXM1, the growth of both cell lines was significantly inhibited at 24 h, with the IC50 being 16.87 μM for JeKo-1 and 13.73 μM for Mino. To confirm the biological importance of FOXM1 in MCL, we employed lentiviral shRNA to suppress FOXM1 expression. As shown in Figure 3B, the cell growth of JeKo-1 and Mino was also significantly decreased at 72 h, with shRNA species #2 more efficient than species #1.

We then performed Western blotting to examine the biochemical changes induced by the suppression of FOXM1 in MCL cells. As shown in Figure 3C, the FOXM1 knockdown efficiency was higher with shRNA species #2 than species #1, and this finding correlates with the results illustrated in Figure 3B. In the same experiment, we found that the shRNA-mediated FOXM1 downregulation led to dramatic decreases in c-Myc, p65 and cyclin D1. In a separate experiment, we also found that shRNA knockdown of FOXM1 in JeKo-1 cells triggered apoptosis, as evidenced by the substantial decreases in Bcl-2 and survivin (two anti-apoptotic proteins) and by the cleavage of PARP and caspase 3 (Figure 3D).

### 3.4. Gene Set Enrichment Analysis Reveals a Link Between FOXM1 and NFκB

**Figure 4:**
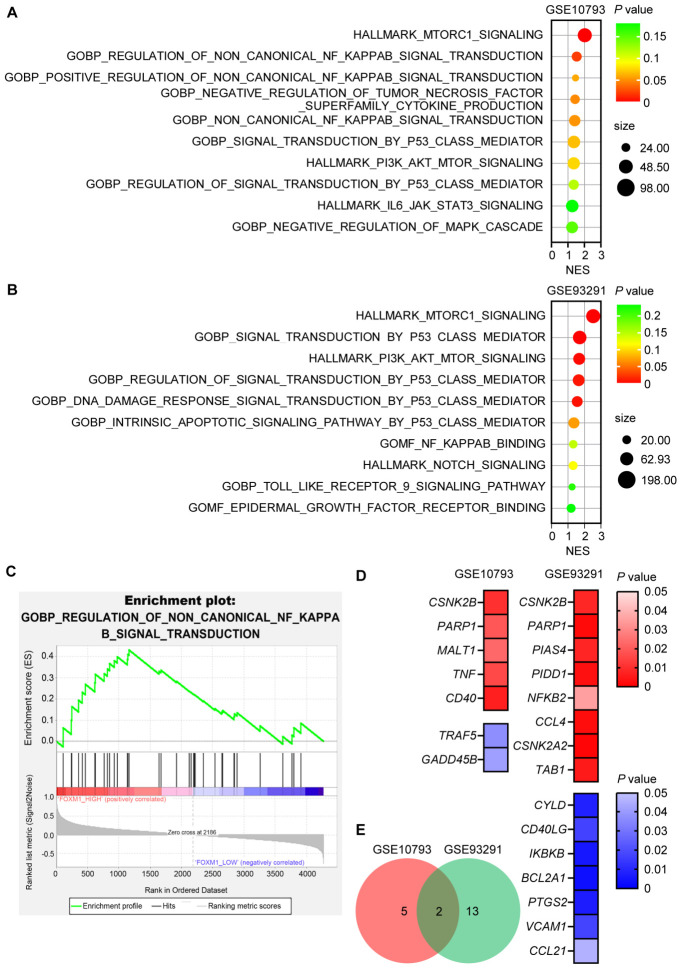
Bioinformatic analysis of the NFκB pathway in two GEO datasets. GSE10793 and GSE93291 datasets were divided into two groups based on the expression level of *FOXM1*. (**A**,**B**) The gene set enrichment analysis results showed the top 10 enriched pathways in the two GEO datasets for the FOXM1 high-expressing group ranked by normalized enrichment score (NES). (**C**) The enrichment plot indicates that the non-canonical NFκB signaling pathway was significantly activated in the *FOXM1* high-expressing group (NES = 1.53, NOM *p*-value = 0.022). (**D**) Correlation analysis between *FOXM1* expression and genes within the NFκB pathway gene set (derived from the KEGG database) was performed using Spearman correlation analysis. *p* values were obtained from correlation tests, and statistical significance was defined as *p* < 0.05. Genes labeled in red are those significantly upregulated in the *FOXM1* high-expressing group, whereas those labeled in blue are significantly downregulated in the same group. (**E**) Venn diagram illustrates that 2 genes were shared between the two datasets.

Using gene set enrichment analysis applied to the same two GEO datasets mentioned above (GSE10793 and GSE93291), we attempted to identify if FOXM1 is functionally linked to the other cellular signaling pathways that have been previously implicated in the pathogenesis of MCL, including those of NFκB, PI3K/Akt, mTOR, NOTCH, JAK/STAT, MEK/ERK, Wnt/β-catenin and toll-like receptors. Our analysis has highlighted the NFκB pathway, as it appeared four times between the two cohorts (Figure 4A,B). Furthermore, NFκB genes were significantly upregulated in the *FOXM1* high-expressing group (Figure 4C). Of the 104 genes belonging to the NFκB pathway, the levels of 20 genes significantly correlate with that of FOXM1 between the two cohorts (Figure 4D,E). Overall, these findings led us to hypothesize that FOXM1 promotes the growth of MCL cells by collaborating with the NFκB pathway, which has been previously reported to be pathogenetically important in MCL [4,35].

### 3.5. FOXM1 Upregulates the NFκB Pathway

**Figure 5:**
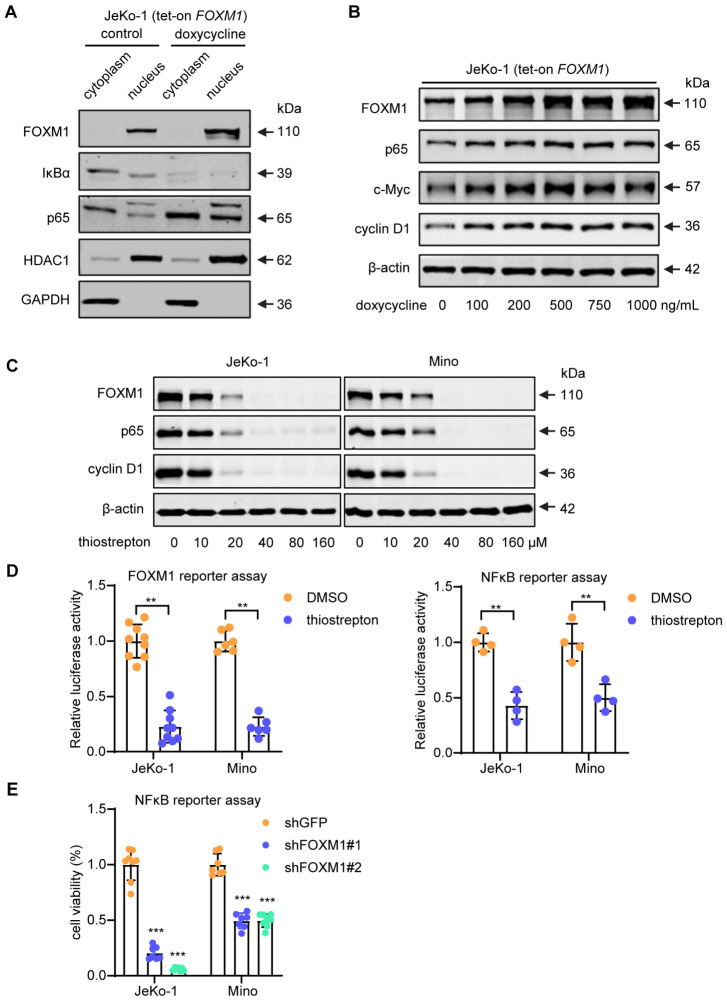
FOXM1 upregulated the NFκB pathway in MCL cells. (**A**) Nuclear/cytoplasmic fractionation of FOXM1, IκBα and p65 in tet-on FOXM1 JeKo-1 cells. The addition of doxycycline to the cell culture resulted in a substantial decrease in IκBα and an appreciable increase in the total and nuclear p65 proteins. HDAC1 and GAPDH were used as the nuclear and cytoplasmic markers separately. (**B**) Western blot analysis using tet-on FOXM1 JeKo-1 cells showed that increasing doses of doxycycline increased the expression of FOXM1, cyclin D1, c-Myc and p65 in a dose-dependent manner. (**C**) Western blot analysis of JeKo-1 and Mino treated with thiostrepton showed the opposite effects. β-actin was used as the loading control. (**D**) NFκB and FOXM1 reporter assay using JeKo-1 and Mino treated with thiostrepton showed a significant decrease in the transcription activity of both FOXM1 and NFκB (mean ± SD, *n* = 4, two-tailed unpaired Student’s *t*-test). (**E**) Luciferase assays of JeKo-1 and Mino treated with *FOXM1* shRNAs led to significant reductions in the transcription activity of NFκB (mean ± SD, *n* = 7, one-way ANOVA with Dunnett’s test vs. shGFP; ** *p* < 0.01, *** *p* < 0.001).

To test our hypothesis, we examined if FOXM1 directly contributes to the activation of the NFκB pathway in MCL. We generated JeKo-1 cells stably transduced with a lentiviral tetracycline-on *FOXM1* expression plasmid (tet-on *FOXM1*). As shown in Figure 5A, using nuclear/cytoplasmic fractionation, enforced expression of *FOXM1* resulted in an appreciable elevation of nuclear p65 (the key transcriptional mediator of the canonical NFκB pathway) and a dramatic downregulation of IκBα, a crucial negative regulator of the canonical NFκB pathway that keeps NFκB factors from entering the nucleus and promotes their proteasomal degradation [36].

As shown in Figure 5B, using the same tet-on FOXM1 cells, the gradual increase in FOXM1 led to the dose-dependent upregulation of the protein levels of p65 as well as two of the downstream targets of NFκB, cyclin D1 and Myc. Conversely, inhibition of FOXM1 using thiostrepton in JeKo-1 and Mino cells led to a dramatic downregulation of p65 in a dose-dependent manner; the downstream target cyclin D1 decreased accordingly (Figure 5C). As shown in Supplemental Figure S1, enforced expression of FOXM1 in tet-on FOXM1 cells led to an appreciable increase in the gene level of CSNK2B, one of the known activators of NFκB [37]. Take together, these findings suggest that, in addition to the downregulation of IκBα, FOXM1 may promote the activation of the NFκB canonical pathway via multiple mechanisms.

To substantiate the functional relationship between FOXM1 and NFκB, we measured the transcriptional activity of NFκB using a luciferase-based reporter assay. As shown in Figure 5D,E, treatment of the two MCL cell lines with thiostrepton or shRNA against FOXM1 led to a significant and dramatic reduction in the NFκB transcriptional activity.

### 3.6. NFκB Upregulates FOXM1 in MCL Cells

**Figure 6:**
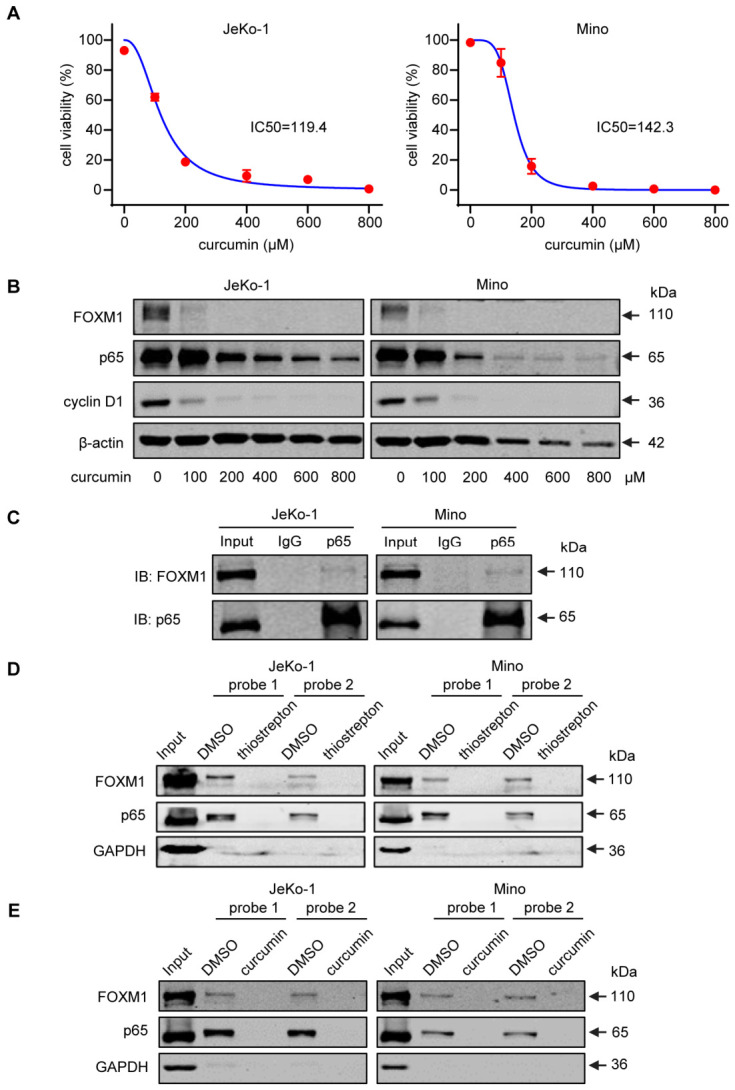
NFκB induces FOXM1 expression by binding its promoter region. (**A**) Cell viability assay of two MCL cell lines treated with the NFκB inhibitor curcumin. Data are presented as mean ± SD (*n* = 5). Dose–response curves were analyzed using nonlinear regression to calculate IC50 values (variable slope, four-parameter logistic model). (**B**) Western blot analysis of p65, cyclin D1 and FOXM1 in JeKo-1 and Mino cells treated with curcumin. β-actin was used as the loading control. (**C**) Co-IP experiments were performed to detect the physical binding of FOXM1 and p65 in MCL cells. (**D**,**E**) Two DNA probes were designed to examine the binding of NFκB and FOXM1 to the FOXM1 promoter; probe 1 and probe 2 contain the NFκB and FOXM1 binding site, respectively. The effects of thiostrepton or curcumin on the DNA binding of these two transcriptional factors are illustrated. DMSO was used as the control.

We then tested if NFκB can upregulate FOXM1, thus forming a positive feedback loop with FOXM1. As shown in Figure 6A, curcumin, a pharmacologic agent commonly used to inhibit NFκB, effectively inhibited the growth of both JeKo-1 and Mino, and this finding aligns with that of a previous study [38]. Correlating with these phenotypic changes, curcumin dramatically decreased the protein expressions of p65 and its downstream target cyclin D1, as well as FOXM1 (Figure 6B).

In further support that NFκB and FOXM1 form a positive feedback loop, we found evidence of physical interaction between p65 and FOXM1 by using Co-IP. As shown in Figure 6C, the use of anti-p65 in Co-IP studies also pulled down a small amount of FOXM1. Of note, the small amount of FOXM1 pulled down by anti-p65 can be partly explained by the fact that only a small fraction of the total cellular p65 protein is localized to the nuclei (Figure 5A).

We then performed DNA pulldown assay using DNA probes containing the consensus binding sequence for NFκB (probe #1) or FOXM1 (probe #2). As shown in Figure 6D, the use of the NFκB probe pulled down an abundant amount of p65 as well as FOXM1. Importantly, the pull-down of p65 was largely abrogated when thiostrepton was added, suggesting that the DNA binding/transcriptional activity of p65 is dependent on its binding to FOXM1. We repeated similar experiments using curcumin instead of thiostrepton, and similar results were obtained (Figure 6E). Taken together, these findings suggest that the binding of p65 and FOXM1 to each other is the optimal configuration for their DNA binding/transcriptional activity.

### 3.7. Overexpression of FOXM1 Partially Overcomes the Inhibitory Effects of Curcumin

**Figure 7:**
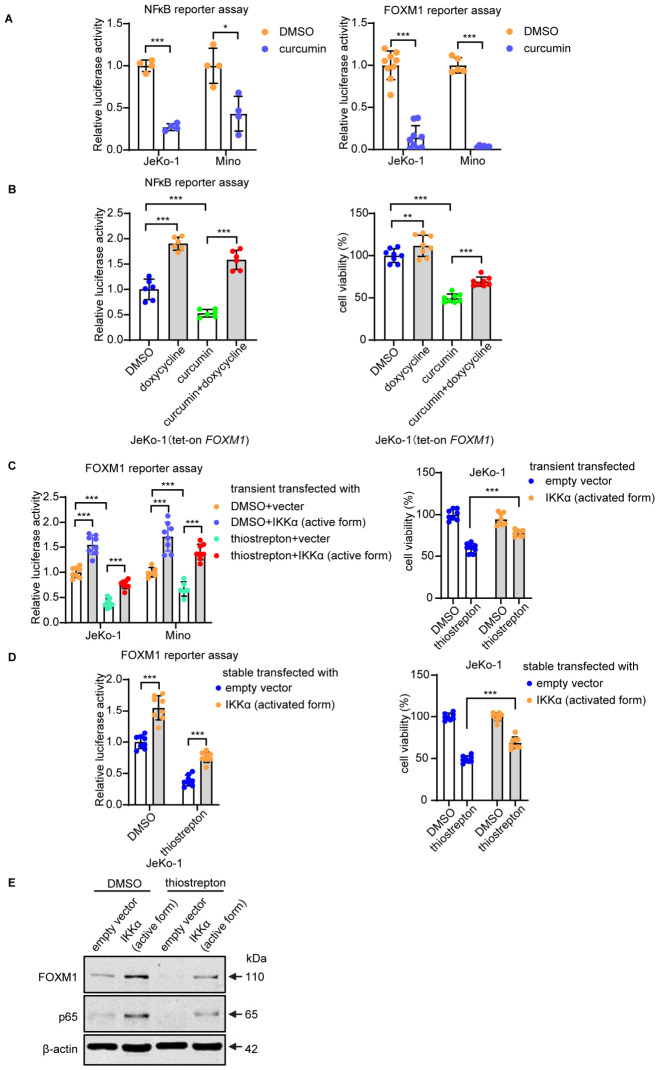
FOXM1 and NFκB activate each other’s transcription activity. (**A**) Luciferase assays of NFκB and FOXM1 reporter activities in JeKo-1 and Mino that were treated with curcumin. DMSO was used as the control (mean ± SD, *n* = 4, two-tailed unpaired Student’s *t*-test). (**B**) Luciferase assay of NFκB reporter activity and cell viability assays in tet-on FOXM1 JeKo-1 cells that were treated with curcumin, doxycycline, or a combination of both drugs (mean ± SD, *n* = 6, two-way ANOVA). (**C**) Luciferase assay of FOXM1 reporter activity and cell viability assays in JeKo-1 or Mino cells that were transient transfected with the indicated plasmids then treated with/without thiostrepton (mean ± SD, *n* = 6, two-way ANOVA). (**D**) Luciferase assay of FOXM1 reporter activity and cell viability assays in JeKo-1 cells that were stably transfected with the indicated plasmids then treated with/without thiostrepton (mean ± SD, *n* = 8, two-way ANOVA). (**E**) Western blot analysis for FOXM1 and p65 in JeKo-1 cells that stably transfected with indicated plasmids then treated with/without thiostrepton. * *p* < 0.05, ** *p* < 0.01, *** *p* < 0.001.

To further illustrate the functional relationship between FOXM1 and NFκB, we asked if overexpression of FOXM1 can help overcome the inhibitory effects of curcumin on the transcription activity and cell-growth effects of NFκB. As illustrated in Figure 7A, the transcriptional activity of NFκB, measured by a luciferase-based reporter assay, was significantly suppressed by curcumin. As shown in Figure 7B, similar curcumin-mediated suppression of the NFκB transcriptional activity in tet-on FOXM1 cells was observed. Importantly, this suppression was significantly overcome when FOXM1 was upregulated by adding doxycycline to the cell culture. Conversely, as shown in Figure 7C–E, activation of the NFκB pathway, achieved by overexpressing a constitutively active IKKα vector, abrogated the thiostrepton-mediated suppressive effect on the transcription activity of FOXM1. The biological effects of using the constitutively active IKKα vector on the expression of p65 and FOXM1 are illustrated in Figure 7E; thiostrepton-treated samples were included for comparison.

### 3.8. Colocalization of FOXM1 and p65

**Figure 8:**
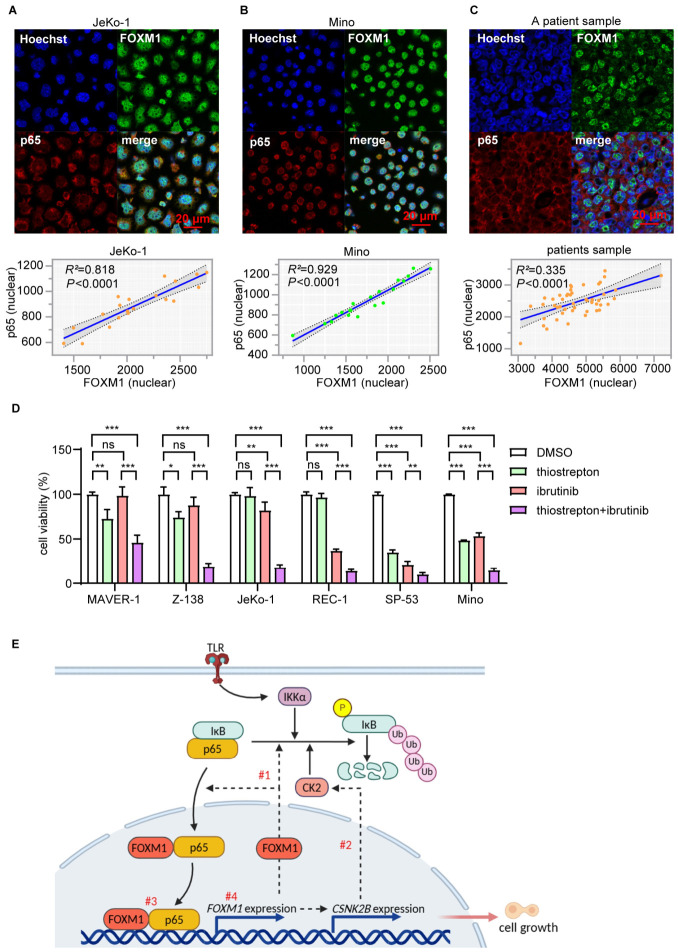
Colocalization of FOXM1 and p65 in MCL cells and patient samples, sensitization of MCL cell lines to ibrutinib by thiostrepton, and schematic model of the FOXM1/NFκB positive feedback loop. (**A**,**B**) Immunofluorescence staining of FOXM1 and p65 in JeKo-1 and Mino cells. Hoechst was used as a nuclear marker. Linear regression analysis with Pearson correlation (two-tailed) was performed to assess the association between nuclear FOXM1 and p65 expression (*R*^2^ and *p* values are shown). Hoechst, blue color; FOXM1, green color; p65, red color. (**C**) Immunofluorescence staining of FOXM1 and p65 in MCL patient samples. Hoechst was used as a nuclear marker. Linear regression analysis and Pearson correlation analysis were performed based on the nuclear protein expression levels of FOXM1 and p65. Bar = 20 μM. Merged images indicate colocalization. Each dot represents one analyzed region. The blue solid line shows the linear regression fit, the grey shaded area indicates the 95% confidence interval, and dashed lines denote the confidence bounds. (**D**) Each cell line was cultured under normal conditions and treated with ibrutinib and/or thiostrepton for 24 h, and cell viability was assessed using the CCK8 assay. The DMSO group served as the control. (mean ± SD, *n* = 3, two-way ANOVA; * *p* < 0.05, ** *p* < 0.01, *** *p* < 0.001.) (**E**) Our data suggest that FOXM1 and NFκB form a positive feedback loop in promoting cell growth of MCL. This collaboration appears to occur at multiple levels. FOXM1 increases NFκB nuclear localization (#1) and its protein level by promoting the proteasomal degradation of IκB, a key negative regulator of this signaling pathway. It is possible that the protein level of p65 is further increased by FOXM1 via other mechanisms. Some of the downstream targets of FOXM1 such as CSNK2B are known activators of the NFκB pathway (#2). The DNA binding and transcriptional activity of p65 are greatly enhanced when it is bound to FOXM1 in the nucleus; the vice versa is true for FOXM1 when it is bound to NFκB (#3). NFκB activation upregulates the expression level of FOXM1 (#4), creating a positive feedback loop. Solid arrows represent direct interactions, while dashed arrows indicate indirect or hypothetical relationships.

To further support the concept that the transcriptional activities of FOXM1 and p65 are dependent on their physical interaction in the nucleus, we performed immunofluorescence double staining and confocal microscopy using MCL cell lines (JeKo-1 and Mino) as well as tumors (*n* = 4). Similar findings were observed across cell lines and tumors, with representative images shown in Figure 8A–C. FOXM1 was found largely in the nuclei of MCL cell lines and tumor cells. In contrast, most p65 was found localized in the cytoplasm. Nevertheless, the fusion signals of FOXM1 and p65 were detected in the nuclei of both MCL cell lines (illustrated in Figure 8A,B) and 4 of 4 patient samples (illustrated in Figure 8C). The expression level of nuclear FOXM1 significantly correlated with that of nuclear p65 on a single-cell level.

### 3.9. Inhibition of FOXM1 Sensitizes MCL Cells to Ibrutinib

It has been previously suggested that the NFκB signaling contributes to ibrutinib resistance in the two ibrutinib-resistant MCL cell lines, MAVER-1 and Z-138 [7]. Since we found evidence that FOXM1 forms a positive feedback loop with NFκB, we hypothesized that inhibition of FOXM1 using thiostrepton can significantly sensitize MCL cell lines to ibrutinib. In this experiment, we included both MAVER-1 and Z-138 cells, as well as 4 other MCL cell lines that are considered ibrutinib-sensitive. At a dose of 10 μM of ibrutinib, we found that MAVER-1, Z-138 and JeKo-1 were relatively resistant to ibrutinib, where the other three cell lines (SP-53, REC-1 and Mino) were relatively sensitive (Figure 8D). At a relatively low dose of thiostrepton (0.6 μM), all 6 cell lines showed significant sensitization to ibrutinib-induced cell-growth inhibition, with the most dramatic sensitization observed in the 3 ibrutinib-resistant cell lines.

## 4. Discussion

FOXM1 has been recognized as an important oncoprotein in many cancer models, but its pathogenetic importance in MCL has not been extensively studied. The first evidence that FOXM1 is biologically important in MCL was previously provided in a poster presentation, in which pharmacological inhibition of FOXM1 using thiostrepton was found to effectively inhibit the growth of MCL cells in vitro and in vivo [27]. This conclusion is in alignment with our observations described in this study. Briefly, we found that inhibition of FOXM1 using thiostrepton or shRNA can effectively inhibit the growth of MCL cells in vitro, and this observation was associated with increased apoptotic activity. Furthermore, using GEO datasets generated from two cohorts of previously untreated MCL patients, we found a significant inverse correlation between the expression of *FOXM1* and the OS of MCL patients. Of note, the prognostic significance of *FOXM1* is independent of that of *MKI67*, suggesting that FOXM1 may mediate its oncogenic effects by involving processes not directly linked to cell proliferation. In this regard, previous studies of FOXM1 using a variety of cancer cell lines have shown that this oncoprotein can interrupt diverse cellular processes by modulating the transcription of many gene targets, and these pathways are involved in the regulation of cell-cycle progression, senescence, apoptosis, genomic stability, cell migration/invasiveness, energy metabolism, response to oxidative stress, cancer stemness and drug resistance [13]. The multi-functionality of FOXM1 has made this protein an attractive anti-cancer therapeutic target, for which various pharmacologic inhibitors have been developed [13].

To decipher the mechanisms by which FOXM1 exerts its oncogenic effects in MCL, we correlated the expression of *FOXM1* with those of several key signaling pathways implicated in the pathogenesis of MCL. Our studies had highlighted the NFκB pathway, whose pathogenetic role in MCL has been recognized since the early 2000s [4]. In the literature, we found only two studies describing the phenomenon that FOXM1 can activate and promote the NFκB pathway in 293T (a human embryonic kidney cell line) and K562 cells (a human chronic myelogenous leukemia cell line), although the underlying mechanisms were not investigated in detail [39,40]. In this current study, we found evidence that FOXM1 and NFκB work collaboratively, and they form a positive feedback loop in promoting the growth of MCL cells. Specifically, we found evidence that FOXM1 in MCL cells can upregulate the protein level and nuclear localization of p65, as well as the DNA binding/transcription activity of the NFκB pathway. Furthermore, enforced expression of FOXM1 using JeKo-1 cells stably transduced with tet-on *FOXM1* can overcome the inhibitory effect of curcumin on the transcription activity of NFκB and the growth of MCL cells. Our data suggest that FOXM1 promotes NFκB signaling at multiple levels, and the model is illustrated in Figure 8E. FOXM1 can increase the total protein level by promoting proteasomal degradation of IκB, the key negative regulator of NFκB. The protein level of p65 also may be increased, either directly or indirectly, owing to the transcriptional regulatory function of FOXM1. For instance, we found that FOXM1 can upregulate CSNK2B, a known activator of the NFκB pathway. The nuclear localization of p65 is increased with the increased degradation of IκB. Lastly, we have provided evidence that the DNA binding/transcriptional activity of p65 is greatly enhanced when it complexes with FOXM1 in the nucleus.

Conversely, we found evidence that NFκB can upregulate FOXM1 and promote its DNA binding/transcription activity. Thus, inhibition of NFκB using curcumin resulted in a dramatic downregulation of FOXM1 and its transcription activity, whereas expression of the constitutively active form of IKKα can significantly diminish the inhibitory effect of thiostrepton on MCL cells. As illustrated in Figure 8E, NFκB promotes the oncogenic effects of FOXM1 via at least two mechanisms. Firstly, the expression level of FOXM1 can be effectively upregulated by NFκB. Secondly, the DNA binding/gene transcription activity of FOXM1 is enhanced by the binding of p65 to FOXM1 in the nucleus. Our hypothetical model is supported by our confocal microscopy study results. This concept also correlates well with the finding of a previously published study in which a substantial co-occupancy of FOXM1 and NFκB was identified on NFκB target genes revealed by large-scale ChIP studies [41]. Of note, it is well established that the aberrant expression of FOXM1 can be mediated by a wide range of molecular defects ranging from FOXM1 gene mutations, activation by other signaling pathways (e.g., K-ras, TNFR-HIFα as well as Ras/Akt), post-transcriptional modifications that can increase the biological activity or stability of FOXM1, abnormal expression of specific miRNA species (e.g., miRNA23a) and various post-translational modifications of FOXM1. Considering the biological significance of FOXM1 in MCL, future studies may focus on delineating the exact mechanisms by which FOXM1 is upregulated in MCL cells.

Our single-cell transcriptomic analysis has revealed that *FOXM1* and *MKI67* are co-expressed at high levels in distinct subsets of tumor cells. In view of the finding that the same cell subsets are highly represented in the relapse tumor samples, it is tempting to speculate that FOXM1 contributes to disease relapses in MCL, which remains to be one of the most significant clinical challenges. Ibrutinib has been used for MCL patients who relapse after the initial conventional combination chemotherapy, and the adoption of ibrutinib has significantly prolonged the OS of MCL patients [42]. Nonetheless, ibrutinib resistance remains a major clinical challenge for MCL patients. While the underlying mechanisms are likely multi-factorial, some of the recent research has highlighted the importance of NFκB in this context [5]. Thus, our identification of FOXM1 as a collaborator of NFκB led us to hypothesize that FOXM1 inhibition is an effective means to lower ibrutinib resistance in relapse MCL. In alignment with this concept, we found the addition of a relatively low dose of thiostrepton can significantly sensitize MCL cells to the inhibitory effects of ibrutinib, and the extent of sensitization is most dramatic in MAVER-1 and Z-138, the two cell lines commonly considered as ibrutinib-resistant.

To our knowledge, all MCL cell lines and tumors used in this study were derived from nodal MCL. Furthermore, since conventional MCL (i.e., nodal MCL with classic morphologic features) is far more common than leukemic MCL, cases included in the GEO datasets used in this study were also mostly derived from nodal MCL (based on the available published information). Thus, we believe that our conclusions regarding FOXM1 and its forming a positive feedback loop with NFκB are only applicable to conventional MCL. Whether FOXM1 is important in leukemic MCL or the blastic variant of MCL needs to be further studied.

## 5. Conclusions

In conclusion, we presented evidence that the oncogenic protein FOXM1 is highly and consistently expressed in MCL, and it promotes the cell growth of MCL. Furthermore, single-cell transcriptomic analysis revealed B cell-enriched populations with high FOXM1 and MKI67 expression that are associated with disease relapse, highlighting a potential cellular context in which FOXM1 may contribute to MCL progression and therapeutic resistance. While FOXM1 is known to mediate its oncogenic effects via its known multi-functionality in cancer cell types, this study has highlighted its collaboration with the NFκB as an important mechanism to promote cell growth and ibrutinib resistance in MCL. We believe that further studies to evaluate FOXM1 as a potential therapeutic target, particularly in the context of fighting ibrutinib resistance in MCL, are warranted.

## Data Availability

The raw data supporting the conclusions of this article will be made available by the authors on request.

## Supplementary Materials

The following supporting information can be downloaded at: https://www.mdpi.com/article/10.3390/cells15090776/s1, Supplemental File S1: Supplemental Materials and Methods, Supplemental Figure S1: FOXM1 upregulates *CSNK2B* expression. References [26,43] has been cited in the supplementary materials.

## Abbreviations

The following abbreviations are used in this manuscript:

shRNA	short hairpin RNA
IKKα	inhibitor of nuclear factor kappa-B kinase subunit alpha
MKI67	marker of proliferation Kiel 67
