# Supplementary material for: FOXM1 and NFκB Form a Positive Feedback Loop to Promote Cell Growth and Drug Resistance in Mantle Cell Lymphoma
Source: Cells. 2026 Apr 25;15(9):776. doi: 10.3390/cells15090776 (PMC13162716; doi:10.3390/cells15090776)
Supplement: Supplementary file 1 [file cells-15-00776-s001.zip › cells-4242690-supplementary.pdf]

Supplemental Material for

FOXM1 and NFκB Form a Positive Feedback Loop to Promote Cell Growth and Drug Resistance in Mantle Cell Lymphoma

Yujie Zhang [1,2], Yuqi Song [2], Meaad Almowaled [3], Chuquan Shang [2], Leizhao Hua [2], Irwindeep Sandhu [4,5], Anthea Peters [4,5], Michael Patvin Chu [4,5], Peng Wang [4,5,*] and Raymond Lai [2,5,*]

[1] Department of Physiology, School of Basic Medical Science, Nanjing Medical University, Nanjing, China.

[2] Department of Laboratory Medicine and Pathology, University of Alberta, Edmonton, Canada.

[3] College of Applied Medical Sciences, King Saud Bin Abdulaziz University for Health Sciences, Jeddah, Saudi Arabia.

[4] Division of Hematology, Department of Medicine, University of Alberta, Edmonton, Canada.

[5] Department of Oncology, Cross Cancer Institute, Edmonton, Canada.

* Address correspondence to: pw2@ualberta.ca (P.W.); rlai@ualberta.ca (R.L.)

Materials and Methods

Plasmids, transfection, and lentiviral transduction

Short hairpin RNA (shRNA) plasmids for *FOXM1* were purchased from Dharmacon. Plasmids of GFP shRNA, doxycycline inducible overexpression of FOXM1B, pcw107, and IKKα (S176E, S180E)-pcw107-V5 were purchased from Addgene (Watertown, MA, USA). Lenti-X 293T cells were seeded in 60 mm dishes and subsequently transfected with the transfer vector, psPAX2, and pMD2.G plasmids (Addgene, Cambridge, MA, USA) using Lipofectamine 2000 (Invitrogen, Carlsbad, CA, USA). After transfection for 48 hours, the viral supernatant was collected for transduction, as described previously[1]. Subsequently, 1×10^6 JeKo-1 cells were incubated with 1 mL of viral supernatant containing 1.2 µg/mL polybrene (Sigma-Aldrich, St. Louis, MO, USA) for each well of a 6-well plate. The plate was then spun at 1000g, 32°C for 2 hours to facilitate viral infection. Following the spin-infection step, 2 mL of fresh medium was added to each well. 24 hours after, cells were washed with PBS, resuspended in fresh medium in T25 flasks and incubated. Subsequently, cells were collected 24 hours later for subsequent assays and Western blot analysis.

Antibodies information

The following antibodies were used: GAPDH (Bioss Antibodies, Woburn, MA, USA, Cat. #bsm-33033m, 1:5000), β-actin (Santa Cruz Biotechnology, Dallas, TX, USA, Cat. #sc-17829, 1:1000), FOXM1 (Santa Cruz Biotechnology, Cat. #sc- 376471, 1:1000 and 1:50), PARP1 (Santa Cruz Biotechnology, Cat. #sc-56196, 1:1000), HDAC1 (Santa Cruz Biotechnology, Cat. #81598, 1:1000), survivin (Santa Cruz Biotechnology, Cat. #sc-10811, 1:1000), p65 (Abcam, Cambridge, MA, USA, Cat. #ab16502, 1:1000 and 1:50), c-Myc (Abcam, Cat. #ab32072, 1:1000), cyclin D1 (Abcam, Cat. #ab16663, 1:1000), IκBα (Cell Signaling Technology, Danvers, MA, USA, Cat. #9242, 1:1000), Bcl-2 (Cell Signaling Technology, Cat. #15071, 1:1000), caspase-3 (Cell Signaling Technology, Cat. #9662, 1:1000), cleaved caspase-3 (Cell Signaling Technology, Cat. #9664, 1:1000), Normal Rabbit IgG (Millipore, Burlington, MA, USA, Cat. #12-370, 1:50), Normal Mouse IgG (Millipore, Cat. #12-371, 1:50), IRDye 800CW Goat anti-rabbit (LI-COR Biosciences, Lincoln, NE, USA, Cat. #926-32211, 1:20000) and anti-mouse IgG secondary antibody (LI-COR Biosciences, Cat. # 926-32210, 1:20000).

Western blot analysis and co-immunoprecipitation

Membranes were washed with TBST and visualized using the Odyssey Western Blot Imager (LI-COR Bioscience, Lincoln, NE, USA).

Quantitative real-time PCR (qPCR)

Total RNAs were extracted from cells using RNeasy Plus Mini Kit (Qiagen, Valencia, CA, USA). Reverse Transcription reactions were performed with 1 μg of total RNA using the High-Capacity cDNA Reverse Transcription Kit (Thermo Fisher Scientific, Cleveland, OH, USA). Quantitative real-time PCR reactions were conducted as described previously[2]. *GAPDH* was used as the loading control. The primers sequences were as follows: *CSNK2B*-F, TGAGCAGGTCCCTCACTATC; *CSNK2B*-R: GTAGCGGGCGTGGATCAAT; *GAPDH*-F: GGAGCGAGATCCCTCCAAAAT; and *GAPDH*-R: GGCTGTTGTCATACTTCTCATGG. The relative expression was determined using the $2^{-\Delta\Delta Ct}$ method.

Immunohistochemistry and immunofluorescence

For MCL cells and 4 MCL patient tumor sections, the immunofluorescence assay utilized an anti-FOXM1 mouse antibody (Santa Cruz Biotechnology, 1: 100) and anti-p65 rabbit antibody (Abcam, 1: 100) were used in the immunofluorescence assay. Hoechst 33342 (Thermo Fisher Scientific, 1:5000) was used to stain cell nuclei. Cells and tissues were visualized with a Zeiss LSM510 confocal microscope (Carl Zeiss, Heidelberg, Germany) using a 40× oil lens at the Core Cell Imaging Facility, Cross Cancer Institute, University of Alberta, Edmonton, Canada. The pixel intensity of the fluorescence image was analyzed using Adobe Photoshop CS6 (Adobe Systems, San Jose, California, USA).

Luciferase assay

The luciferase reporter assay was performed using a Luciferase Assay System kit (Promega Corporation, Madison, USA), according to the manufacturer’s protocol. In brief, cells were transiently transfected with FOXM1 luciferase reporter plasmid or NFκB luciferase reporter plasmid. Cells were lysed with a passive lysis buffer followed by estimation of protein concentration. Equal amounts of protein for each sample were then assessed for luciferase activity using the FLUOstar Omega multi-mode microplate reader (BMG Labtech, Ortenburg, Baden-Wurttemberg, Germany) to read and analyze data.

DNA pull-down

The sequences of the probes were designed based on the promoter sequence of FOXM1, with the consensus site underlined as follows, probe1 (p65): CCTCCCGGGATCCCCCGGGGTT, probe2 (FOXM1): TGCCACTGTATAAATACAATAC. Mutant DNA probes were used as negative controls to optimize the protocol. Following addition of the biotinylated probes and Streptavidin agarose beads (40 µL, Thermo Fisher Scientific), the mixture was then incubated with rotation for 2 hours at room temperature. The samples were then collected by centrifugation at 550×g and the supernatants were discarded. The beads were then washed three times with ice cold PBS with protease inhibitors. Protein elution was achieved by boiling the beads at 100°C in the 2× Sample loading buffer, before loading proteins onto SDS-PAGE gels.

Supplemental Figure S1 and Legend

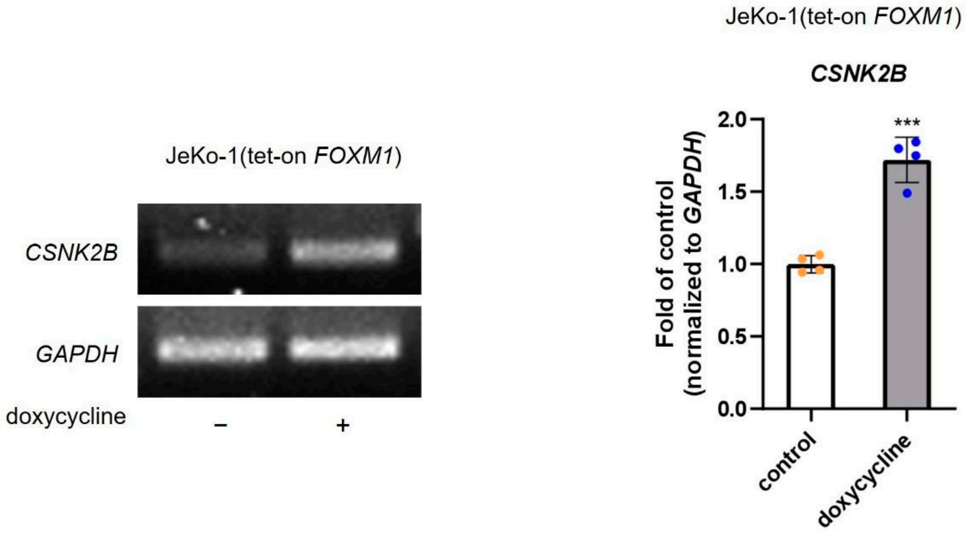

Supplementary Figure S1. FOXM1 upregulates *CSNK2B* expression.

Reverse transcription PCR was performed to assess the effect of FOXM1 on the gene expression of *CSNK2B* in JeKo-1 cells stably transduced with tet-on *FOXM1*. The addition of doxycycline to the cell culture significantly upregulated the *CSNK2B* gene level (left panel). Similar results were obtained when quantitative PCR were performed (right panel). ***$P < .001$

Supplemental References

1. Haque, M.; Li, J.; Huang, Y.-H.; Almowaled, M.; Barger, C.J.; Karpf, A.R.; et al. NPM-ALK is a key regulator of the oncoprotein FOXM1 in ALK-positive anaplastic large cell lymphoma. *Cancers (Basel)* **2019**, *11*, 1119.
2. Wu, C.; Molavi, O.; Zhang, H.; Gupta, N.; Alshareef, A.; Bone, K.M.; et al. STAT1 is phosphorylated and downregulated by the oncogenic tyrosine kinase NPM-ALK in ALK-positive anaplastic large-cell lymphoma. *Blood* **2015**, *126*, 336–345.
